# FGF21 Requires βklotho to Act *In Vivo*

**DOI:** 10.1371/journal.pone.0049977

**Published:** 2012-11-27

**Authors:** Andrew C. Adams, Christine C. Cheng, Tamer Coskun, Alexei Kharitonenkov

**Affiliations:** Lilly Research Laboratories, Lilly Corporate Center, Indianapolis, Indiana, United States of America; Wageningen University, The Netherlands

## Abstract

FGF21 has gradually become a focal point in metabolic research given its intriguing and complex biology and relevance to drug discovery. Despite the large amount of accumulated data, there remains a dearth of understanding of FGF21 physiology at the molecular/whole organism level. The scaffold protein βklotho (KLB) has previously been demonstrated *in vitro* to function as a co-factor permitting FGF21 mediated FGF receptor activation. However, the requirement for KLB in the propagation of FGF21 action in living animals has yet to be evaluated. To answer this question, we tested FGF21 in mice with total body ablation of KLB (KLBKO) and found no detectable activity. Firstly, we demonstrate that the disruption of KLB entirely abrogates acute FGF21 signaling in adipose tissue. We go on to show that this signaling defect translates to the absence of FGF21 mediated metabolic improvements in DIO mice. Indeed, KLBKO mice are totally refractory to FGF21-induced normalization of glucose homeostasis, attenuation of dyslipidemia, elevation of energy expenditure and weight loss. The lack of FGF21-driven effects was further substantiated at the transcriptional level with no FGF21 target gene signature detectable in adipose tissue and liver of KLBKO animals. Taken together our data show that KLB is a vital component of the FGF21 *in vivo* signaling machinery and is critically required for FGF21 action at the whole organism level.

## Introduction

The identification of FGF21 as a metabolic regulator has triggered extensive study in part due to the therapeutic potential of FGF21 to treat metabolic disease [1], [2]. FGF21 is a member of the “hormone-like” sub group of the FGF superfamily [3], [4], a classification which was made due to its inability to bind heparin [1], [5] and presence in circulation in animals [6], [7] and man [8]. In place of heparin, the transmembrane protein Klotho Beta (KLB) is thought to facilitate FGF21 activation of FGFRs thus determining the tissue specificity of FGF21 action [5], [9], [10] and mediating the variety of FGF21s metabolic actions [1].

Strong evidence exists that FGF21 binds KLB and that this interaction is a necessary step in FGF21 receptor complex activation *in vitro* [5], [9]–[12]. While the role of FGFR1 as the major FGF21 receptor *in vivo* has recently become apparent [13], the requirement of KLB in propagation of FGF21 metabolic actions in animals has yet to be demonstrated with earlier reports even suggesting that FGF21 is able to function in a KLB independent manner both *in vitro* [14] and *in vivo* [15].

In the present study we sought to clarify the extent to which KLB is required for the function of FGF21 in animals. To achieve this goal we generated mice with whole body deletion of KLB (KLBKO) and subjected them to the acute and chronic treatment with FGF21. The outcomes of this investigation are indicative that FGF21 cannot induce acute signaling, act on its target genes or produce integrated metabolic effects in KLB-deficient animals. Taken together our data show that KLB is a necessary co-factor for FGF21 action *in vivo*.

## Materials and Methods

### Generation of KLBKO Mice

KLB mutant mice (KLBKO) were generated utilizing the FLP/FRT recombination system by Taconic-Artemis (Cologne, Germany). Exons 1–4 of the mouse KLB gene were flanked with FRT sites. After administration of hormones, superovulated BALB/c females were mated with BALB/c males. Blastocysts were then isolated from the uterus at day 3.5. A flat tip, piezo actuated microinjection-pipette was used to inject targeted C57BL/6NTac ES cells into each blastocyst. After recovery, injected blastocysts were transferred to each uterine horn 2.5 days post coitus of pseudopregnant NMRI females. Chimerism was measured in chimeras (G0) by coat color contribution of ES cells to the BALB/c host (black/white). Highly chimeric mice were bred to C57BL/6-Tg (CAG-Flpe) females to excise the targeted region. Genotype was assessed using PCR with the following primer sequences (CAACTATAGCAGCTGAGACTGACG and ACAAATCTTCAGTCGGTGGTG). Prior to study mice were crossed onto the C57BL/6 background for at least 10 generations.

### Protein Production

FGF21 was generated as previously described [16].

### Animals

All animals were individually housed in a temperature-controlled (24°C) facility with 12 h/12 h light/dark cycle. Animal protocols in this study were approved by the Eli Lilly and Co. Animal Use and Care Committee (Protocol No. 12225).

### Acute Signaling Studies

Male WT and KLBKO mice (n = 4 per time point) were maintained on a standard chow diet (TD2014; Harlan Teklad, Madison, WI) prior to treatment. To determine acute signaling responses mice were administered 1 mg/kg of recombinant human FGF21 and then sacrificed at specified time points.

### FGF21 Treatment of Diet Induced Obese WT and KLBKO Mice

Male WT and KLBKO mice (n = 5 per group) were maintained on a calorie-rich diet consisting of 40% fat, 39% carbohydrate, and 21% protein caloric content (TD95217; Harlan Teklad, Madison, WI) and had free access to food and water before randomization by weight. Mice were administered FGF21 for a period of 14 days via continuous infusion using osmotic mini-pumps (ALZET, Cupertino, CA) at dose of 1 mg/kg/day. Prior sacrifice glucose levels were determined using Precision G Blood Glucose Testing System (Abbott Laboratories, Abbott Park, IL). Following sacrifice tissues were rapidly dissected and flash frozen in liquid nitrogen.

### Analysis of Metabolites and Circulating Factors

Blood samples were collected on ice temperature prior to storage of plasma at −80°C. Serum metabolites were measured by small-scale enzymatic assays for glucose, β-hydroxybutyrate, triglycerides, and cholesterol (Stanbio Laboratory). Insulin (Crystal Chem Inc.), leptin (Crystal Chem Inc.) and Total Adiponectin (BioVendor Inc.) were measured by specific ELISA.

### RNA Isolation, RT and Real-time Quantitative PCR

RNA was isolated from tissues using TRIzol reagent (Invitrogen, Carlsbad, CA) or by homogenization of frozen samples in Lysing Matrix D shaker tubes (MP Biomedicals, Santa Ana, CA) and was reverse transcribed into cDNA using a High-Capacity cDNA Reverse Transcription Kit (PE Applied Biosystems, Foster City, CA). Reactions were performed in triplicate on an ABI Prism 7900HT (PE Applied Biosystems) and were normalized to PPIA mRNA.

### Immunoblotting

Immunoblotting of WAT and liver extracts from WT and KLBKO mice was performed using an anti-KLB (R&D Systems), and β-actin as a loading control (Sigma).

### Statistical Analysis

Data are presented as mean±SEM. Statistical analysis was performed using one-way ANOVA, followed by Dunnett’s multiple comparisons test where appropriate. Differences were considered significant when P = <0.05.

## Results

**Figure 1:**
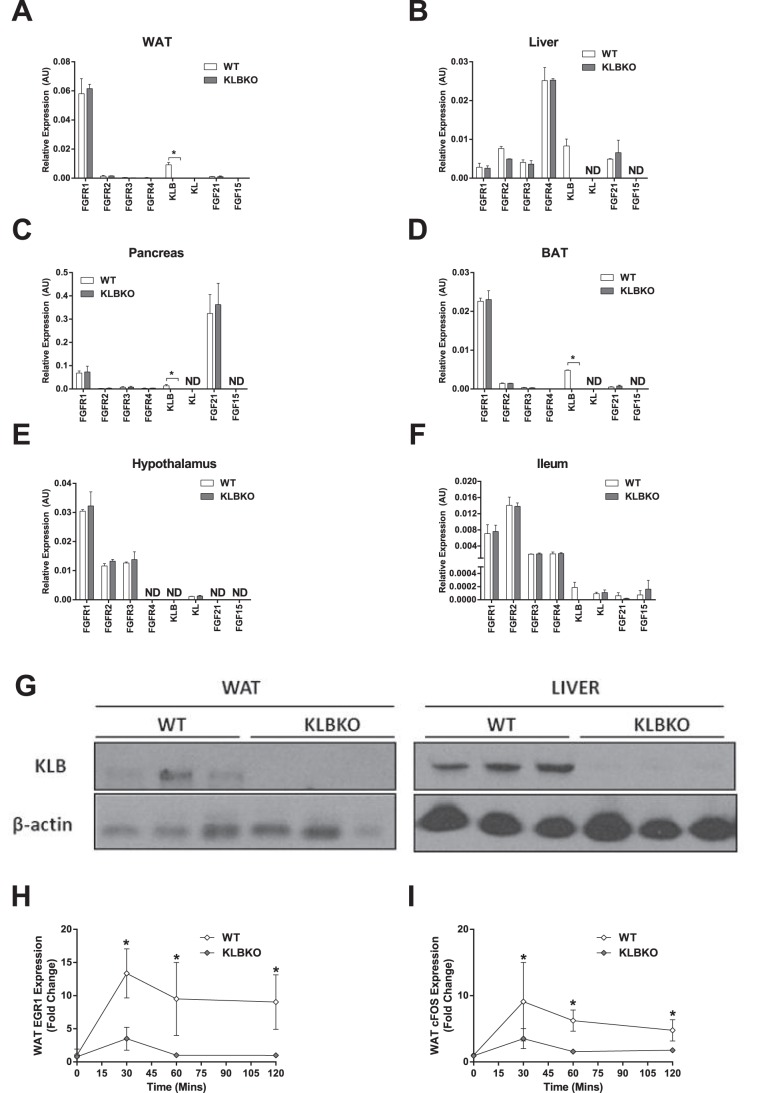
Expression of FGFR/Klotho receptor components (A–F), KLB protein expression in liver and WAT (G). Induction of immediate early genes EGR-1 (H) and cFOS (I) expression in WAT of WT and KLBKO animals following acute FGF21 treatment. Statistical analysis was performed using one-way ANOVA, followed by Dunnett’s multiple comparisons test where appropriate. Differences were considered significant when P≤0.05.

We first investigated levels of KLB transcript in WT and KLBKO mice in tissues which have been suggested to have detectable KLB expression, namely, WAT, BAT, liver, pancreas, hypothalamus, and ileum (Figure 1A–F; [17]), and presence of KLB protein in WAT and liver (Figure 1G). We also profiled these tissues for the mRNA expression of all four FGFR variants, αKlotho (KL; [18]), another member of Klotho family, FGF21, and FGF15, the putative mouse ortholog of human FGF19, which is known to signal via the KLB/FGFR complex and also through FGFR4 alone (Figure 1A–F; [12]). While KLBKO mice were indeed devoid of KLB expression at the transcriptional and protein levels in all tissues tested, the mRNA expression levels of FGFR1-4, FGF21, FGF15, KL were essentially identical between WT and KLBKO mice (Figure 1A–G).

Following administration of exogenous FGF21 we examined acute signaling in WAT, a tissue we and others have recently shown to be the primary mediator for the metabolic actions of FGF21 *in vivo* [13], [19]. Supportive of the hypothesis that KLB is explicitly required to trigger a response to FGF21, we found no statistically significant EGR1 or cFOS activation in the WAT of the KLBKO animals while in WT mice FGF21 treatment induced a robust effect (Figure 1 H&I).

When maintained on either chow or HFD for a period of 8 weeks the KLBKO mice were indistinguishable from WT counterparts with regards to food intake, energy expenditure (EE) and fed serum glucose indicative that at baseline the impact of FGF21/FGFR1/KLB axis on energy homeostasis is masked. These findings are reminiscent of the phenotype observed in the FGF21KO [20] and FR1KO mice [13] which require a metabolic challenge in the form of HFD feeding or exposure to an environmental stressor to become evident.

**Figure 2:**
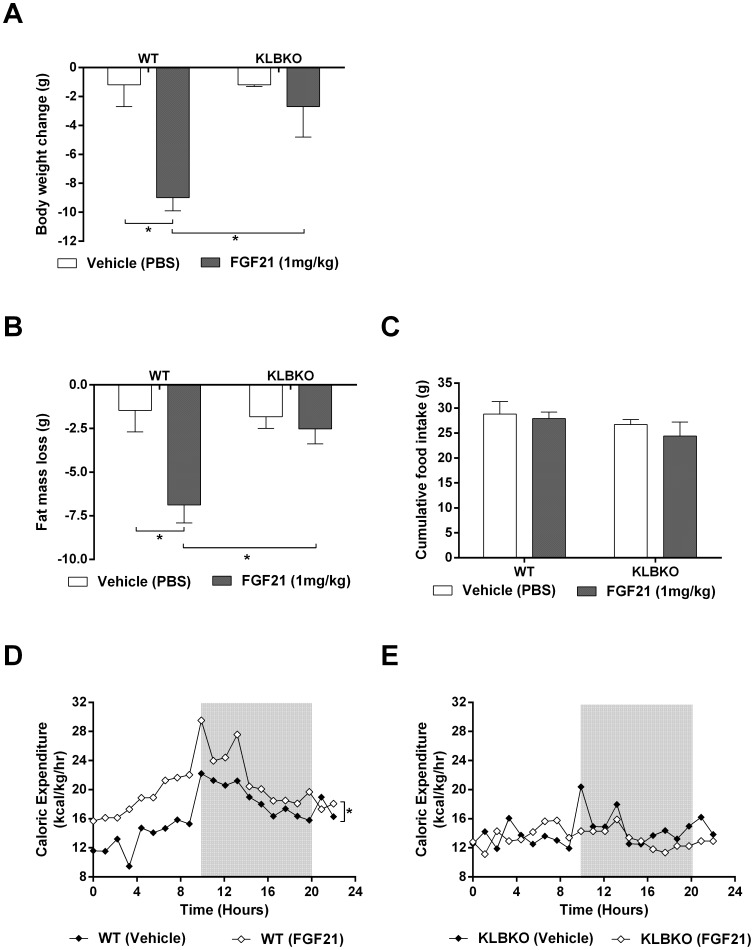
Following chronic FGF21 treatment we assessed weight loss (A), fat mass loss (B), food intake (C) and energy expenditure in WT (D) and KLBKO mice (E). Statistical analysis was performed using one-way ANOVA, followed by Dunnett’s multiple comparisons test where appropriate. Differences were considered significant when P≤0.05.

**Figure 3:**
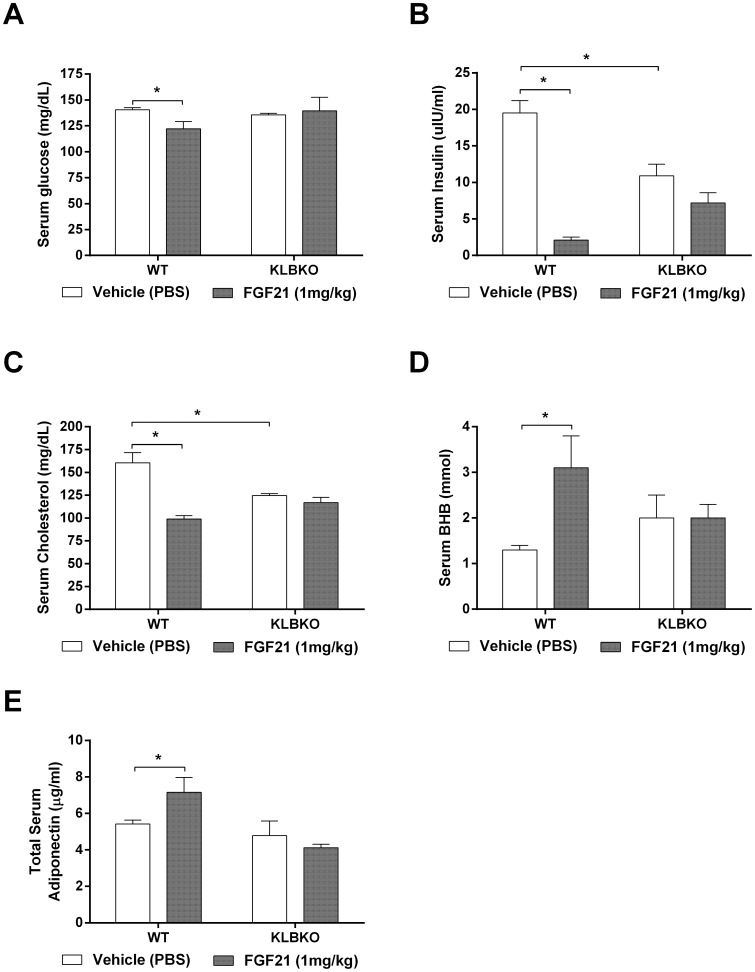
To determine the effect of chronic FGF21 on circulating hormones and metabolites we measured plasma glucose (A), insulin (B), cholesterol (C), β-Hydroxybutyrate (D), adiponectin (E). Statistical analysis was performed using one-way ANOVA, followed by Dunnett’s multiple comparisons test where appropriate. Differences were considered significant when P≤0.05.

Given the absence of acute FGF21 signaling we went on to examine the metabolic effects of chronic FGF21 administration. Consistent with the established anti-obesity action of FGF21 [12], [21], [22], WT animals exhibited sustained weight loss of approximately 9 g which was significantly attenuated in the KLBKO mice (Figure 2A). As we and others have reported previously [19], [21], the FGF21-induced reduction in body mass was primarily driven by improvements in total adiposity in the WT mice; again no effects were noted in the KLBKO group (Figure 2B). Furthermore, WT animals treated with FGF21 exhibited an increase in energy expenditure (Figure 2D), however, this phenomenon was absent in the KLBKO cohort (Figure 2E). FGF21 treatment had no effect on total caloric intake in either genotype (Figure 2C) confirming earlier observations [11] with this data being further supportive of the hypothesis that FGF21 mediated weight loss is driven by increased metabolic rate. Finally, in WT animals FGF21 significantly lowered serum glucose, insulin and cholesterol, and increased ketone body production as measured by βHB in plasma, however, all of these known pharmacologic effects of FGF21 [12], [13], [23] were not present in the KLBKO mice (Figure 3 A–D).

We have recently suggested that FGF21-driven FGFR1 engagement of adipose tissue [13] and resultant adipokine secretion [24] are critical aspects of the *in vivo* mechanism of FGF21 action. Confirming this hypothesis we saw an increase in serum adiponectin (Figure 3E) in WT but not KLBKO mice following FGF21 administration. Plasma leptin, another adipokine known to be regulated by FGF21 [12], [19], [25], was decreased in WT but KLBKO mice (Data not shown).

To determine if the differences in chronic FGF21 effects between the WT and KLBKO mice correlated with transcriptional changes we examined gene expression in WAT, BAT and liver. In WAT of WT animals we found increases in known FGF21 target genes including LEPR, PGC1α and UCP1 [19], [21], [26], but KLBKO mice lacked these effects (Table 1). Interestingly, UCP1 mRNA was significantly reduced at baseline in the WAT of the KLBKO mice suggesting a role for KLB/FGF21 in regulation of basal UCP1 expression in this tissue. The FGF21 gene expression signature including increased expression of ACC1, ACC2, UCP1 and PGC1α were present in the BAT of WT mice, however, there were no significant changes in the mRNA levels of these genes in the KLBKO animals (Table 2).

**Table 1:** White adipose tissue.

	Wild type		KLBKO	
	Vehicle(PBS)	FGF21(1 mg/kg)	Vehicle(PBS)	FGF21(1 mg/kg)
**LEPR**	1 (0.01)	1.70 (0.39)*	1.28 (0.50)	0.95 (0.05)
**PGC1α**	1 (0.01)	1.51 (0.09)*	1.08 (0.14)	1.23 (0.41)
**UCP1**	1 (0.49)	2.49 (0.88)*	0.40 (0.15)*	0.20 (0.05)*

To determine if the transcriptional events downstream of KLB/FGFR activation were altered we examined gene expression in white adipose tissue (WAT). Data are presented as mean±SEM in brackets. Statistical analysis was performed using one-way ANOVA, followed by Dunnett’s multiple comparisons test where appropriate. Differences were considered significant when P≤0.05.

**Table 2:** Brown adipose tissue.

	Wild type		KLBKO	
	Vehicle(PBS)	FGF21(1 mg/kg)	Vehicle(PBS)	FGF21(1 mg/kg)
**ACC1**	1 (0.01)	1.55 (0.04)*	1.03 (0.15)	0.95 (0.12)
**ACC2**	1 (0.01)	1.64 (0.01)*	0.93 (0.15)	0.91 (0.10)
**PGC1α**	1 (0.01)	1.49 (0.23)*	1.11 (0.08)	0.99 (0.08)
**UCP1**	1 (0.11)	1.51 (0.35)*	1.03 (0.10)	0.91 (0.10)

To determine if the transcriptional events downstream of KLB/FGFR activation were altered we examined gene expression in brown adipose tissue (BAT). Data are presented as mean±SEM in brackets. Statistical analysis was performed using one-way ANOVA, followed by Dunnett’s multiple comparisons test where appropriate. Differences were considered significant when P≤0.05.

In the liver FGF21 induced expression of leptin receptor and lowered SCD1 in WT but not in KLBKO mice, suggesting improvements in leptin sensitivity and the presence of leptin receptor/SCD1 interplay only in the WT animals (Table 3, [13], [21]). Consistent with previously published report [13] FGF21 had no effect on CYP7a1 expression in either genotype. Of potential importance, the expression of CYP7a1 was increased and SCD1 lowered in a basal state in the KLBKO mice when compared to WT. Therefore, based on gene expression analysis on WAT, BAT and liver we can conclude that the presence of KLB is an explicit requirement for FGF21 mediated transcriptional activity in these tissues.

**Table 3:** Liver.

	Wild type		KLBKO	
	Vehicle(PBS)	FGF21(1 mg/kg)	Vehicle(PBS)	FGF21(1 mg/kg)
**CYP7a1**	1 (0.62)	1.07 (0.49)	2.82 (0.01)*	3.18 (0.45)*
**LEPR**	1 (0.12)	2.31 (0.65)*	0.56 (0.05)	0.67 (0.15)
**SCD1**	1 (0.06)	0.15 (0.05)*	0.61 (0.01)*	0.62 (0.11)*

To determine if the transcriptional events downstream of KLB/FGFR activation were altered we examined gene expression in Liver. Data are presented as mean±SEM in brackets. Statistical analysis was performed using one-way ANOVA, followed by Dunnett’s multiple comparisons test where appropriate. Differences were considered significant when P≤0.05.

## Discussion

We have recently demonstrated that adipose tissue is the main target organ which serves to mediate the majority of FGF21 pharmacology *in vivo* [13]. Using animals with adipose-specific ablation of FGFR1, we showed that these mice failed to respond to FGF21 treatment spanning an array of signaling responses, gene expression and integrated pharmacology outputs. Thus, this work along with earlier observations [19], [27], [28] establishes FGFR1 in adipose as the main receptor/tissue which underlies the multitudes of beneficial FGF21 pharmacology.

Even though FGF21 was initially shown to directly activate FGFRs at supra-pharmacologic doses [14] later work decisively established the role of KLB in place of heparin as a necessary co-factor to trigger the FGF21 signal *in vitro* [5], [10], [11], [28]. Indeed, KLB and FGFRs have been shown to form a constitutive FGF21 receptor complex, in which KLB functions as a scaffold to allow FGF21 bind the receptor complex and the FGFR represents an activity-competent element to propagate the signal downstream [5]. We have previously demonstrated that the FGF21 ΔN17 mutant, a competitive KLB antagonist is able to block FGF21’s glycaemic effects [29] suggestive that KLB is required to propagate FGF21’s signal *in vivo*. The hypothesis that KLB represents an integral component of FGF21 receptor has previously never been directly tested in animals, with all of the current evidence derived from tissue culture studies [11], [28], [30]. Furthermore, there is even a potential controversy suggestive of either a poor translation of *in vitro* findings into *in vivo* physiologic outcomes, or confounding methodological discrepancies, given that the initial publication on mice lacking KLB claimed that FGF21 signaling was fully preserved in KLBKO mice [15], while more recent studies arrive at a completely opposite conclusion [12], [27]. In order to resolve this controversy we decided to generate our own KLB deficient animals and extend the *in vivo* FGF21 tests in these animals beyond basic signaling readouts as previously reported [15], [27].

Importantly, while analyzing these mice, we found that aside from a lack of KLB, the distribution of FGF21, Klotho, FGF15, and FGF receptors remains unchanged in several tissues in KLBKO mice as compared to WTs (Figure 1A–F). Coupled with the absence of a basal phenotype due to KLB ablation, this finding makes our KLBKO animals a suitable model to investigate the role of KLB for FGF21 action *in vivo*.

At the signaling level we show that removal of KLB abrogates the ability of FGF21 to activate WAT in a manner found in WT animals (figure 1G–H) indicative that KLB is absolutely required for receptor engagement and subsequent early transcriptional activation. This result comes opposite to the conclusion in Tomiyama *et al* (2010) but consistent with Yang *et al* (2012). We suggest that the differences between our study and Tomiyama *et al* (2010) may be due to the quality/purity of protein utilized in these experiments. Indeed, Tomiyama *et al* (2010) employed non-purified supernatants from FGF21 cDNA transfected cells while the test article used in this study and others [27] was purified FGF21 protein.

Coupled with the signaling response there was a profound FGF21-induced shift in energy homeostasis but only in WT animals. Furthermore, FGF21 mediated effects on weight loss and glucose/lipid control in WT mice were all absent in the KLBKO animals. Thus, lack of WAT engagement at the signaling level due to KLB ablation translates to a complete inability of FGF21 to affect metabolic endpoints (Figure 2 A–E). Interestingly, serum insulin was significantly reduced at baseline in the KLBKO animals, a difference which may be due to effects of FGF21 on insulin production in the pancreas [31].

Of note, the metabolic effects of FGF21 in WT mice were largely reflected at the transcriptional level with significant induction of “browning” related genes in WAT such as UCP1 and PGC1α (Table 1), a phenomenon which has recently become synonymous with the mode of action of FGF21 *in vivo* [19], [26], [32], [33]. It is important to note that UCP1 is lower in the WAT of KLBKO mice and is unchanged by FGF21 treatment (Table 1). This observation is especially critical given the fact that there is no decrease in energy expenditure in the vehicle treated KLBKO mice when compared to WT, indirectly suggesting that the elevation of UCP1 in WAT may not be a driver of FGF21 mediated increases in EE. Furthermore, there is no change in UCP1 mRNA in BAT and no difference in EE in KLBKO at baseline. Following an FGF21 challenge, however, UCP1 increased in BAT from WT but not KLBKO animals (Table 2). These data suggest that FGF21 driven activation in BAT [33] rather than in WAT causes elevated energy expenditure, an idea supported by the other recent publication on KLB ablation [34].

In the liver cholesterol 7α-hydroxylase (CYP7A1), a rate-limiting enzyme responsible for conversion of cholesterol to bile acids, plays a key role in regulating lipid and bile salt homeostasis and a key target gene for FGF19 action *in vivo* [28]. Patients presenting with genetic defects in the CYP7A1 exhibit marked hyperlipidemia, including hypercholesterolemia [35]. Ablation of CYP7a1 in mice also results in hypercholesterolemia [36]. Therefore it was interesting to note an increase in hepatic CYP7a1 expression in KLBKO mice at baseline (Table 3) similar in magnitude to the earlier report [15]. This increase was concomitant with a significant reduction in circulating levels of cholesterol (Figure 3B) with both, CYP7a1 expression and plasma cholesterol levels, being unaffected by FGF21 in KLBKO mice. Since in WT animals FGF21 does not regulate CYP7a1 ([28]; Table 3) the FGF21 cholesterol lowering effect is not CYP7a1 mediated but still requires KLB as it is lost in KLBKO mice.

SCD1 (Table 3), which we have recently shown to be a direct target of FGF21 [13] was also reduced in the KLBKO liver at baseline. Upon FGF21 treatment a dramatic reduction in SCD1 expression was observed in the WT animals but there was no FGF21 effect on SCD1 in the KLBKO mice (Table 3). Thus, the perturbation of the hepatic FGF21 axis can lead to altered basal expression of SCD1 and further confirms the idea that this gene is a direct target of FGF21 downstream of the FGFR1/KLB complex.

In parallel with our work, an independent group has recently communicated the metabolic phenotype of both whole body and adipose specific KLB null mice [34]. In this manuscript Ding *et al* (2012) describe severely attenuated signaling in KLBKO mice following FGF21 treatment similar in magnitude to the effect we communicate in this report. However, to study the role of KLB in the majority of their FGF21 metabolic studies a cross of KLBKO mice to FGF21 transgenic (FGF21Tg) mice was utilized [33], rather than injections of recombinant FGF21 as in our study. Thus, due to the potential impact of enhanced action of KLB and FGF21 during development, Ding *et al*’s (2012) results may not be directly comparable to our more discrete pharmacologic studies. Nevertheless, in the whole body KLBKO/FGF21Tg mice deletion of KLB significantly impairs the efficacy of FGF21 [33], the same conclusion we reach in this report.

Furthermore, Ding and colleagues (2012) also conditionally deleted KLB from adipose. Analogous to our recent report on FGFR1 adipose null mice [13], animals with fat-specific KLB ablation lacked insulin sensitization and lipid lowering effects of FGF21. Importantly, the increase in adiponectin observed with either FGF21 treatment in rodents and primates or in FGF21 transgenic mice [13], [16], [19], [24], [37] was absent in adipose specific KLB null mice, further positioning this important insulin sensitizer as a critical downstream mediator of FGF21 action *in vivo*. Loss of KLB [34] led to similar changes at the transcriptional level to those reported in the present study with impaired induction of thermogenic genes in WAT and BAT and the absence of reduced SCD1 in liver. Finally, the body weight phenotype observed in FGF21Tg mice is also lost when KLB is deleted [33], suggesting that this effect in the basal state is mediated by activation of the traditional KLB dependent signaling pathway during development.

Taken as a whole our data span the gamut of FGF21 biology from acute signaling to the integrated metabolic effects at the whole body level and demonstrate the ultimate need of KLB in supporting the action of FGF21 in living animals. Coupled with our earlier data on adipose specific deletion of FGFR1 [13] and the recent report detailing specific deletion of KLB [34] we contend that the FGF21 receptor complex mediating major aspects of FGF21 metabolic effects comprises both, KLB and FGFR1, and resides in the adipose tissue. These data represent a significant advance in the understanding the molecular aspects of FGF21 *in vivo* action and present with the new developmental opportunities to design novel therapeutics that target FGF21 pathway.
